# Erratum

**DOI:** 10.4269/ajtmh.2012.873err

**Published:** 2012-09-05

In *Am J Trop Med Hyg* 84:575–580 by Fabbro et al, one of the author's names is misspelled. The sixth author's name should be Iván Marcipar, not Mancipar. The journal regrets this error.

